# Glycine Receptor Activation Impairs ATP-Induced Calcium Transients in Cultured Cortical Astrocytes

**DOI:** 10.3389/fnmol.2017.00444

**Published:** 2018-01-17

**Authors:** Tatiana P. Morais, David Coelho, Sandra H. Vaz, Ana M. Sebastião, Cláudia A. Valente

**Affiliations:** ^1^Faculdade de Medicina, Universidade de Lisboa, Lisbon, Portugal; ^2^Instituto de Medicina Molecular, Faculdade de Medicina, Universidade de Lisboa, Lisbon, Portugal

**Keywords:** astrocytes, calcium transients, glycine receptor, inhibitory effect, chloride

## Abstract

In central nervous system, glycine receptor (GlyR) is mostly expressed in the spinal cord and brainstem, but glycinergic transmission related elements have also been identified in the brain. Astrocytes are active elements at the tripartite synapse, being responsible for the maintenance of brain homeostasis and for the fine-tuning of synaptic activity. These cells communicate, spontaneously or in response to a stimulus, by elevations in their cytosolic calcium (calcium transients, Ca^2+^T) that can be propagated to other cells. How these Ca^2+^T are negatively modulated is yet poorly understood. In this work, we evaluated GlyR expression and its role on calcium signaling modulation in rat brain astrocytes. We first proved that GlyR, predominantly subunits α2 and β, was expressed in brain astrocytes and its localization was confirmed in the cytoplasm and astrocytic processes by immunohistochemistry assays. Calcium imaging experiments in cultured astrocytes showed that glycine (500 μM), a GlyR agonist, caused a concentration-dependent reduction in ATP-induced Ca^2+^T, an effect abolished by the GlyR antagonist, strychnine (0.8 μM), as well as by nocodazole (1 μM), known to impair GlyR anchorage to the plasma membrane. This effect was mimicked by activation of GABA_A_R, another Cl^-^-permeable channel. In summary, we demonstrated that GlyR activation in astrocytes mediates an inhibitory effect upon ATP induced Ca^2+^T, which most probably involves changes in membrane permeability to Cl^-^ and requires GlyR anchorage at the plasma membrane. GlyR in astrocytes may thus be part of a mechanism to modulate astrocyte-to-neuron communication.

## Introduction

Astrocytes are key active participants in brain functions (Sofroniew, 2010), being responsible for the maintenance of brain homeostasis (Allen and Barres, 2009), for the fine-tuning of synaptic activity (Araque et al., 1999; Perea and Araque, 2005), and presenting a dynamic role in the bidirectional communication with neurons (Volterra and Steinhäuser, 2004). Astrocytes respond to neurotransmitters released by neurons generating an increase in their intracellular calcium concentration ([Ca^2+^]_i_), a response that can be limited to one astrocyte or propagated to other astrocytes, as a wave of rise in cytosolic calcium, mostly resulting of endoplasmic reticulum Ca^2+^ mobilization (Fellin and Carmignoto, 2004; Perea et al., 2009; Perea and Araque, 2010; Allaman et al., 2011; Volterra et al., 2014). Calcium transients (Ca^2+^T) in astrocytes play a relevant role in intercellular communication at the synapse, since they promote the release of signaling molecules, that may act at sites distant from the initial excitation zone, leading to neuromodulation (Perea and Araque, 2005; Perea et al., 2009; Perea and Araque, 2010; Volterra et al., 2014).

A physiological trigger of astrocytic calcium waves is adenosine-5′-triphosphate (ATP), which is released by neurons and glia cells and plays a central role in astrocyte-to-astrocyte signaling, as well as in astrocyte-to-neuron communication (Burnstock et al., 2011). The role of ATP in intercellular communication results mainly from activation of G-protein coupled P2Y receptors, leading to activation of phospholipase C (PLC), with the associated production of inositol 1,4,5 trisphosphate (IP_3_) and the activation of IP_3_ receptors at the endoplasmic reticulum (ER), resulting in a rise in cytoplasmic calcium levels due to Ca^2+^ release from internal stores (Barres, 2008; Orellana and Stehberg, 2014). Activation of ionotropic receptors permeant to Ca^2+^ can also induce an increase in [Ca^2+^]_i_ (Volterra et al., 2014).

γ-amino butyric acid (GABA) and glycine, play a major role in inhibitory neurotransmission in the CNS. Glycine has a dual role since it acts as an inhibitory neurotransmitter through glycine receptor (GlyR) chloride (Cl^-^) channels, and is a co-agonist of glutamate at ionotropic *N*-Methyl-D-aspartate (NMDA) receptor (NMDAR) (Johnson and Ascher, 1987; Eulenburg et al., 2005; Xu and Gong, 2010; Eulenburg, 2011). As a neurotransmitter, the preponderant role of glycine has been associated to the spinal cord and brainstem, but glycinergic transmission related elements have also been identified in the brain (Danglot et al., 2004; Aroeira et al., 2011, 2014).

Astrocytes are the primary source of hippocampal glycine (Xu and Gong, 2010). The existence of functional GlyR in glial cells of the rat spinal cord has been known for a long time (Kirchhoff et al., 1996). However, there is no evidence of GlyR expression or function in brain astrocytes. The purpose of this work was to assess GlyR expression in cortical astrocytes and evaluate their involvement in ATP-induced Ca^2+^T in these cells.

## Materials and Methods

### Animals

Sprague-Dawley rats were obtained from Charles River (Barcelona, Spain). The European Union guidelines (2010/63/EU) and Portuguese law were respected in all procedures regarding the protection of animals for scientific purposes, being the number of animals and their suffering minimized. This study was approved by the “iMM’s Institutional Animal Welfare Body – ORBEA-iMM and the National competent authority – DGAV (Direção Geral de Alimentação e Veterinária).”

### Reagents and Drugs

Unless otherwise indicated, all reagents were acquired from Sigma (St. Louis, MO, United States).

Adenosine-5′-triphosphate was from Invitrogen (Waltham, MA, United States). α-Cyclopiazonic Acid (CPA) a reticulum Ca^2+^ ATPases inhibitor was from Tocris (Avonmouth, United Kingdom). Dimethylsulfoxide (DMSO), muscimol (a GABA_A_R agonist), Nocodazole (which interferes with microtubules polymerization) and strychnine (a GlyR antagonist) were from Sigma. Gabazine (GABA_A_R antagonist) and glycine (GlyR agonist) were purchased from Abcam (Cambridge, MA, United States). *N,N′*-[1,2-ethanediylbis(oxy-2,1-phenylene)]bis[*N*-[2-[(acetyloxy)methoxy]-2-oxoethyl]]-, bis[(acetyloxy)methyl] (BAPTA-AM) was obtained from Molecular Probes (Eugene, OR, United States). Fura-2 acetoxymethyl ester (Fura-2AM) was from Calbiochem (Darmstadt, Germany).

Nocodazole stock solution (10 mM) was prepared in 1% of DMSO. All aliquots were stored at -20°C until further use. Working aqueous dilution was prepared at the day of the experiment. This drug was used in calcium imaging experiments. The maximum amount of DMSO present in the bath (0.0001% v/v) was devoid of influence in astrocytic calcium transients, when compared with controls (*p* > 0.05).

### Antibodies

The primary antibodies used in western blotting were: mouse monoclonal antibody anti- Glyceraldehyde 3-phosphate dehydrogenase (GAPDH, Abcam, Cambridge, United Kingdom, 1:1000), mouse monoclonal anti-GlyR (mAb4a) (Synaptic Systems, 1:250), rabbit polyclonal anti-gephyrin (Synaptic Systems, 1:500) and goat polyclonal anti-glycine receptor β subunit (Santa Cruz Biotechnology, Santa Cruz, CA, United States 1:200). The secondary antibodies used in the immunoblots were goat anti-mouse, goat anti-rabbit and donkey anti-goat, all IgG-horseradish peroxidase conjugated (Santa Cruz Biotechnology, 1:10,000). All antibodies were diluted in 3% of BSA in TBST.

For immunofluorescence assays the primary antibodies used were mouse monoclonal (1:250) and rabbit polyclonal (1:500) against glial fibrillary acidic protein (GFAP), commonly used as a marker for astrocytes (Wang and Bordey, 2008; Oberheim et al., 2012), both from Sigma. Rabbit polyclonal against GlyRα2 subunit (1:100) was from Santa Cruz. The antibodies against GlyR (1:250), GlyRβ subunit (1:50) and gephyrin (1:100), were the same used for western blotting. The secondary fluorescent-labeled antibodies used were goat anti-mouse Alexa 568, rabbit anti-goat Alexa 568 and goat anti-rabbit-Alexa 488 (Invitrogen, Grand Island, NY, United States, 1:400). All antibodies used in immunohistochemistry assays were diluted in 10% of FBS in PBS, except for mAb4a antibody, which was diluted in 0.25% of gelatin in PBS. The primary antibodies were used in other works from the group (Aroeira et al., 2011, 2014, 2015). Furthermore, the specificity of mAb4a and anti-gephyrin was recently testified (Nakahata et al., 2017), as well as that for anti-GlyRα2 and anti-GlyRβ subunits (García-Alcocer et al., 2008).

### Primary Cultures of Astrocytes

Cultures were prepared as before (Aroeira et al., 2014, 2015). Briefly, astrocyte enriched cultures were prepared from neonatal Sprague-Dawley rat pups cerebral cortex (0–2 days). Animals were sacrificed by decapitation and the brains were dissected in ice cold phosphate buffered saline solution (PBS) (NaCl 137 mM, KCl 2.7 mM, Na_2_HPO_4_.2H_2_O 8 mM and KH_2_PO_4_ 1.5 mM, pH 7.4). Cells were then dissociated in 4.5 g/l glucose Dulbecco’s Modified Eagles Medium (DMEM) (Gibco, Paisley, United Kingdom), supplemented with 10% fetal bovine serum (FBS) (Gibco) and 1% antibiotic/antimycotic. Cell suspension was filtered successively through a 230 μm and a 70 μm (BD Falcon, NJ, United States) cell strainers and centrifuged at room temperature (RT) at 200 *g* for 10 min. The final pellet was ressuspended in 4.5 g/l glucose DMEM, and cells were seeded according to the required assay.

Cultures were kept at 37°C in a humidified atmosphere (5% CO_2_) and medium was changed twice a week. At 10 days *in vitro* (DIV), to remove any contaminating microglia cells and obtain astrocytic-enriched cultures, plates were shaken for 5 h in an orbital shaker at 300 rpm, as previously described (Aroeira et al., 2014).

### Western Blotting

For western blot cells were seeded into 60-mm dishes, and at 10, 14, and 18 DIV cell lysates were obtained.

Lysates were run on a 12% sodium dodecyl sulfate-polyacrylamide gel electrophoresis (SDS-PAGE) to study changes in protein expression. Proteins were transferred to a PVDF membrane (Bio-Rad, Hercules, CA, United States) by electroblotting, and blocked with 3% bovine serum albumin in TBS-T (Tris base 20 mM, NaCl 137 mM and 0.1% Tween-20). Membranes were subsequently incubated with the primary (4°C, overnight) and secondary antibodies (RT, 1 h). Development of protein signal intensity was made with ECL Plus Western Blotting Detection System (Amersham-ECL Western Blotting Detection Reagents from GE Healthcare, Buckingamshire, United Kingdom) and visualized with the ChemiDoc^TM^ XRS+Imager system (Hercules, CA, United States). The integrated intensity of protein bands was calculated using computer assisted densitometry with ImageJ software 1.48 V and standardized for GAPDH levels. Protein levels at 14 and 18 DIV were normalized to 10 DIV levels.

### qPCR

Astrocytes were seeded into 60-mm dishes as for western blotting. The RNA was isolated using QIAGEN RNeasy Mini Kit (Qiagen, Hilden, Germany) and quantified with Nanodrop 1000 (ND-1000 Spectrophotometer, Thermo Scientific). Total RNA (3 μg) was used to synthesize the first-strand of cDNA, in the presence of SuperScript II Reverse Transcriptase (EC 2.7.7.49, Invitrogen, Carlsbad, CA, United States), according to manufacturer’s guidelines (SuperScript First Strand Synthesis Systems for RT-PCR from Invitrogen).

The reaction was carried out in a thermocycler (MyCycler – Bio-Rad, Hercules, CA 94547, United States). cDNA amplification was performed in a Rotor-Gene 6000 real-time rotary analyser thermocycler (Corbett Life Science, Hilden, Germany), using a SYBR Green Master Mix (Applied Biosystems, Foster City, CA, United States) and 0.2 μM of each gene primer. The PCR profile consisted in an initial denaturation for 2 min at 95°C, followed by 50 cycles of 30 s at 94°C, 90 s at 60°C and 60 s at 72°C. The melting curve analysis, used to evaluate the specificity of the reactions, and the cycle threshold (CT) were obtained using the Rotor-gene 6000 Software 1.7 (Corbett, Life Science). Relative quantification was measured by the comparative Pfaffl method (Pfaffl, 2001). β-Actin was used as the internal reference gene in all reactions and replica reactions were always made. Furthermore, two types of negative controls were made: a reaction with cDNA obtained in the absence of SuperScript II and a second one without cDNA.

The primers used were: 5′-AGCCATGTACGTAGCCATCC-3′ and 5′-CTCTCAGCTGTGGTGGTGAA-3′ for β-actin; 5′-ACTCTGCGATTCTACCTTTGG-3′ and 5′-ATATTCATTGTAGGCGAGACGG-3′ for GlyRα1; 5′-CAGAGTTCAGGTTCCAGGG-3′ and 5′-TCCACAAACTTCTTCTTGATAG-3′ for GlyRα2; 5′-CTGTTCATATCAAGCACTTTGC-3′ and 5′-GGGATGACAGGCTTGGCAG-3′ for GlyRβ (Aroeira et al., 2011, 2014, 2015), all purchased from Invitrogen.

### Immunostainings

Detection of GlyR subunits, gephyrin and GFAP was performed by immunohistochemistry assays, in brain slices, as well as in cultured astrocytes.

Brains from 12 weeks-old rats were used. At the day of the experiment, rats were deeply anesthetized with a mixture of Ketamine (120 mg/kg) (Imalgene^®^ 1000 Merial, France) and Xylazine (16 mg/Kg) (Rompun^®^ Bayer, Germany) at 1 mL/kg body weight by intraperitoneal injection (i.p.). When reaching the profound anesthesia state, rats were perfused transcardially with 0.9% saline solution, followed by 4% paraformaldehyde (PFA) in phosphate buffer (pH 7.4), as described before (Gage et al., 2012). After perfusion, animals were decapitated, brains were removed and post-fixed by immersion in 4% PFA overnight at 4°C. After a quick wash in PBS, brains were successively immersed in a 15 and 30% sucrose solution at 4°C. After gelatin embedding (7.5% gelatin in 15% sucrose), brains were sliced at 12 μm of thickness on a cryostat (LEICA CM 3050S, Wetzlar, Germany) by iMM’s Histology and Comparative Pathology Laboratory. Coronal sections, at the level of hippocampus (-2.92 mm and -5.04 mm from Bregma) were collected and then mounted on SuperFrost^®^ Plus slides (Menzel-Glaser, Braunschweig, Germany). The obtained slices were stored at -20°C until further use. For protein detection, slices were washed in PBS for 10 min at 37°C, surrounded with DAKO pen (Dako, Denmark) and again washed with PBS. After 10 min of 0.1 M glycine incubation, slices were permeabilized for 10 min with 0.1% Triton X-100 in PBS. For GlyR detection, sections were subsequently immersed in fresh methanol (10 min at -20°C) and washed twice with PBS. After 3 h of blocking, in 10% FBS in PBST, slices were incubated with the primary antibodies (4°C overnight), and with the fluorescent-labeled secondary antibodies (90 min at RT), diluted in blocking solution. Nuclei staining was made with Hoechst 33342 (1:100 dilution in PBS, Invitrogen) for 10 min at RT and the preparations were mounted in Mowiol.

For cultured astrocytes, cells were seeded on poly-D-lysine hydrobromide (PDL) (25 μg/ml) coated 24-well plates and maintained for 18 days. Cells were fixed with 4% PFA in PBS for 15 min at RT at 10, 14 and 18 DIV, incubated for 10 min in 0.1 M glycine and permeabilized (0.1 % Triton X-100 in PBS) for 10 min. The subsequent protocol was identical to the one performed in brain slices, except for blocking and secondary antibodies’ incubation, which were carried out for 1 h.

### Image Aquisition

Images were acquired on an inverted widefield fluorescence microscope (Zeiss Axiovert 200, Zeiss, Germany), with a monochrome digital camera (AxioCamMR3, Zeiss), using a 40x objective (Zeiss). AxioVision 4 software (Carl Zeiss Imaging Systems, Zeiss) was used for image acquisition. Images were obtained with a frame size of 1388 × 1040 pixels and an object space of 0.25 μm/pixel.

### Calcium Imaging

For calcium imaging in astrocytes (Jacob et al., 2014), cell suspension was plated on glass bottom cell culture dishes (Nest Scientific, Rahway, NJ, United States) coated with 10 μg/ml PDL. At the day of the experiment (12–18 DIV) cells were incubated at 22°C for 45 min with the Ca^2+^ sensitive fluorescent dye fura-2 acetoxymethyl ester (Fura-2AM; 5 μM; Calbiochem, Darmstadt, Germany). After three washes with artificial cerebrospinal fluid (aCSF: NaCl 125 mM, KCl 3 mM, NaH_2_PO_4_ 1.25 mM, CaCl_2_ 2mM, MgSO_4_ 2 mM, D(+)-glucose 10 mM and HEPES 10 mM; pH 7.4 adjusted with NaOH) cells were positioned on an inverted epifluorescent optics microscope (Axiovert 135TV, Zeiss, Germany) with a xenon lamp and band-pass filters of 340 and 380 nm wavelengths. Throughout all experiments, cells were continuously perfused with aCSF (with or without added drugs) at 1.5 ml/s and visualized with a 40x oil-immersion objective (Zeiss). ATP was used as the stimulating agent and was applied through a micropipette placed under visual guidance over a single astroglial cell. ATP was released from the micropipette by focal pressure (10 psi for 200 ms) through a Toohey Spritzer Pressure System Ile (Toohey Company, Fairfield, NJ, United States).

**FIGURE 1:**
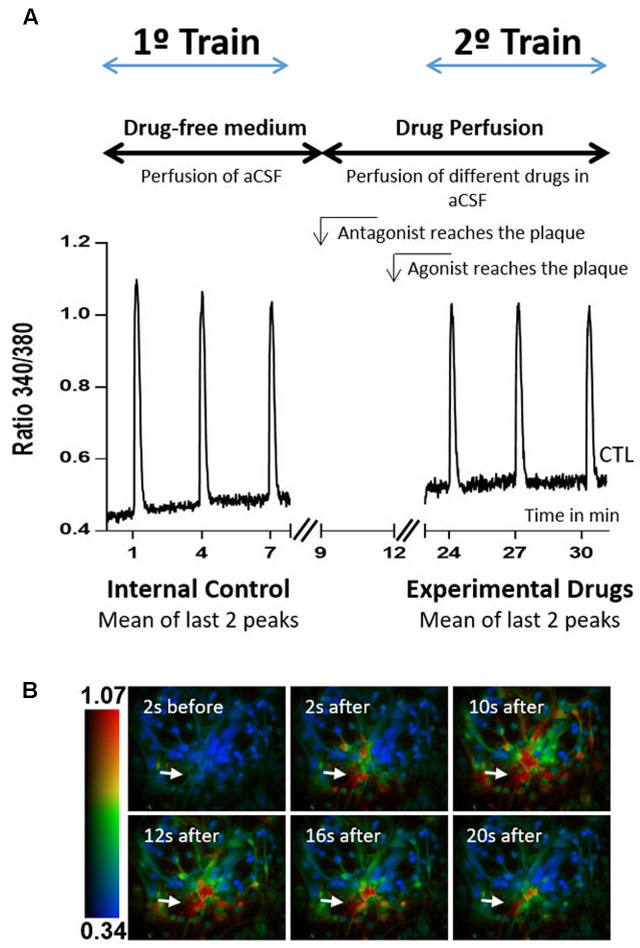
Calcium imaging protocol. **(A)** Protocol design and representative plot responses obtained in one control experiment, i.e., when neither agonists nor antagonists were added to the perfusion solution. The time of arrival of the drug-containing solutions is also indicated. **(B)** Representative images taken before and after exposure to 10 μM ATP, at the times indicated in each panel. The color code refers to the fluorescence ratio 340 nm/380 nm, with higher ratio reflecting higher [Ca^2+^]_i_. Arrows represent the local of ATP pressure application.

Two stimulation trains, with three stimulations each, were conducted in all experiments. The experimental design is represented in **Figure 1**. During the first train, which served as internal control, cells were stimulated with ATP at minutes 1, 4, and 7 after starting the recordings. Stimulation and recording were then stopped and started again at minute 23, ATP stimulations being at minutes 24, 27, and 30. Responses to the first stimulus in each train were discarded and the amplitude of the 2^nd^ and 3^rd^ stimulations were averaged to quantify the response to ATP in each train. Test drugs were added to the perfusion to start reaching the culture dish at min 12 (receptor agonists) or min 9 (receptor antagonists). Drugs were then present in the 2^nd^ train of ATP stimulation and their effects were calculated as the ratio between the amplitudes of ATP response in the 2^nd^ train over that in the 1^st^ train (ratio 2^nd^/1^st^). Drug effects were then compared with the ratio 2^nd^/1^st^ obtained in the absence of any drugs (control), which in this work was 0.89 ± 0.017, *n* = 36.

The responses were recorded by a ratiometric method, in which image pairs were obtained every 250 ms by exciting the preparations at 340 and 380 nm. Fura-2 has an absorbance at 340 nm if bound to calcium, and at 380 nm if not, while the emission wavelength is maintained at 510 nm. The magnitude of the changes in the emission fluorescence of Fura-2 were taken as a measure of the changes in intracellular calcium concentration (Ca^2+^T amplitude), as response to ATP stimulation.

Excitation wavelengths were changed through a high-speed wavelength switcher, Lambda DG-4 (Sutter Instrument, Novato, CA, United States). An estimation of intracellular Ca^2+^ concentration was given by the ratio between the emissions derived from the two excitation wavelengths (340/380). All data was recorded by a cooled CCD camera (Photometrics CoolSNAP) and analyzed using the MetaFluor software (Universal Imaging, West Chester, PA, United States) (Jacob et al., 2014). Regions of interest were acquired by delineating the profile of the cells and averaging the fluorescence intensity inside the delineated area. Peak amplitude was calculated by subtracting the baseline level to the maximum peak intensity.

### Statistical Analysis

Statistical significance was evaluated through the GraphPad Prism version 6 for Windows, GraphPad Software (San Diego, CA, United States). Data are expressed as mean ± SEM from N independent cultures. In calcium imaging experiments the number of responsive cells is designated by n. One-way analysis of variance (ANOVA), followed by Bonferroni’s Comparison Test, was used. Values of *p* ≤ 0.05 were considered to represent statistic differences.

## Results

### GlyR and Gephyrin Are Expressed in Cortical Cultures of Astrocytes

In neurons, GlyR is composed by five protein subunits: α1, α2, α3, α4 (48–49 kDa), and β (58 kDa) (Langosch et al., 1988; Bowery and Smart, 2006; Dutertre et al., 2012). Gephyrin is the protein responsible for GlyR clustering in the post-synaptic membrane, through β subunit binding (Tyagarajan and Fritschy, 2014). We thus assessed the expression of GlyR subunits and gephyrin in cultured astrocytes.

**FIGURE 2:**
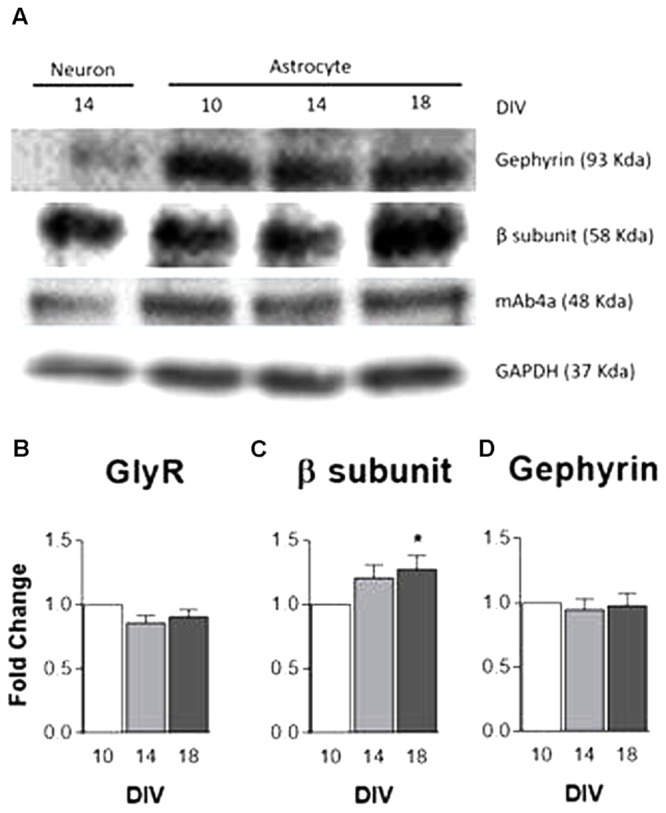
GlyR and gephyrin protein expression. Analysis of GlyR expression in rat primary cultures of cortical astrocytes by western blotting at 10, 14, and 18 DIV. **(A)** Analysis of mAb4a, GlyR β subunit and gephyrin immunoreactivity in total lysates of astrocytes and neurons. Densitometric analysis of **(B)** mAb4a, **(C)** GlyR β subunit **(D)** and gephyrin. GAPDH was used as internal control. The densitometric analysis was performed with the ImageJ software. All values are mean ± SEM, *N* = 3–8 independent cultures. ^∗^*p* ≤ 0.05, one-way ANOVA followed by Bonferroni’s Comparison Test. Statistical tests were performed in comparison to 10 DIV.

The expression levels of GlyR, GlyR β subunit and the GlyR anchoring protein, gephyrin, were evaluated by western blotting with protein extracts from primary cultures of astrocytes at 10, 14, and 18 DIV. GAPDH served as the internal control. Homogenates of cultured neurons were used as a positive control and demonstrate the specificity of the antibodies used. In astrocytes, as illustrated in the immunoblot (**Figure 2A**), the antibodies detected a band of similar molecular weight to that found in neurons.

Densitometric analysis data (**Figures 2B–D**) show a tendency for an increase in GlyR β subunit expression in astrocytes throughout culture time, the increase being significant (*p* < 0.05) at 18 DIV (**Figure 2C**). GlyR (**Figure 2B**) and gephyrin (**Figure 2D**) expression levels persisted nearly constant through time in culture. These results suggest that, in culture, cortical astrocytes express components of the glycinergic synapse, being this the first evidence ever for GlyR expression in astrocytes.

### GlyR α1, α2 and β Subunit mRNA Are Expressed in Cortical Cultures of Astrocytes

**FIGURE 3:**
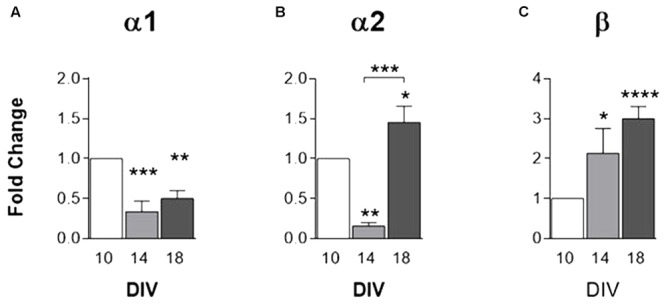
mRNA expression of GlyR subunits. GlyR subunits **(A)** α1, **(B)** α2 and **(C)** β transcripts levels, evaluated by qPCR, in rat cortical astrocytic cultures at 10, 14, and 18 DIV. All values are mean ± SEM, *N* = 3–8 independent cultures. ^∗^*p* ≤ 0.05, ^∗∗^*p* ≤ 0.01 ^∗∗∗^*p* ≤ 0.001, ^∗∗∗∗^*p* ≤ 0.0001, one-way ANOVA followed by Bonferroni’s Comparison Test. Statistical tests were performed in comparison with 10 DIV, except if otherwise indicated by the lines above the bars.

GlyR α1, α2 and β subunits in cultured astrocytes were assessed by qPCR. Data reveal that mRNA expression of GlyR α1 subunit (**Figure 3A**) undergoes a significant decrease within time in culture. GlyR α2 mRNA expression (**Figure 3B**) depicts a significant decrease from 10 to 14 DIV and upsurges at 18 DIV. GlyR β subunit mRNA expression (**Figure 3C**) displays a progressive increase with time in culture.

### GlyR Is Localized Mostly within the Cytosol of Cultured Astrocytes

**FIGURE 4:**
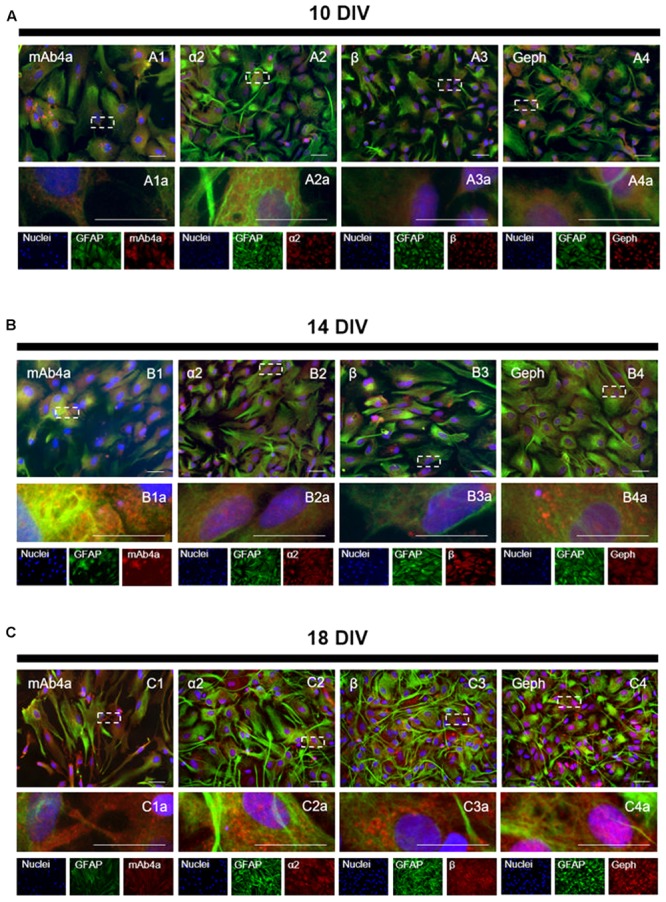
GlyR localization in rat cortical astrocytes. Double detection of GFAP and mAb4a/α2/β/gephyrin in astrocytic cultures, at **(A)** 10, **(B)** 14 and **(C)** 18 DIV. Nuclei were stained with Hoechst, GFAP stained astrocytes are green and mAb4a/α2/β/gephyrin (Geph) immunoreactivity is red. Fluorescence images were acquired with a 40x objective in a Zeiss Axiovert 200. Dotted lines represent the amplified areas. Scale bars, 50 μm.

The subcellular localization of GlyR (stained with mAb4a antibody), GlyR α2 and β subunits, as well as of gephyrin, was investigated through an immunocytochemistry assay (**Figure 4**), in which a double staining of GFAP (astrocytic marker) and mAb4a, GlyR α2 subunit, GlyR β subunit or gephyrin was carried out. Hoechst was used as the nuclear marker. The images acquired show immunostaining for GlyR (mAb4a, **Figures 4A1,B1,C1**), α2 (**Figures 4A2,B2,C2**) and β subunits (**Figures 4A3,B3,C3**), as well as gephyrin (**Figures 4A4,B4,C4**), in cultured astrocytes in all timepoints evaluated. Immunostaining of GlyR and its subunits was observed in the perinuclear space and cytoplasm, while gephyrin immunostaining was detected mostly around the nucleus.

### GlyR Is Expressed in Rat Brain Astrocytes

**FIGURE 5:**
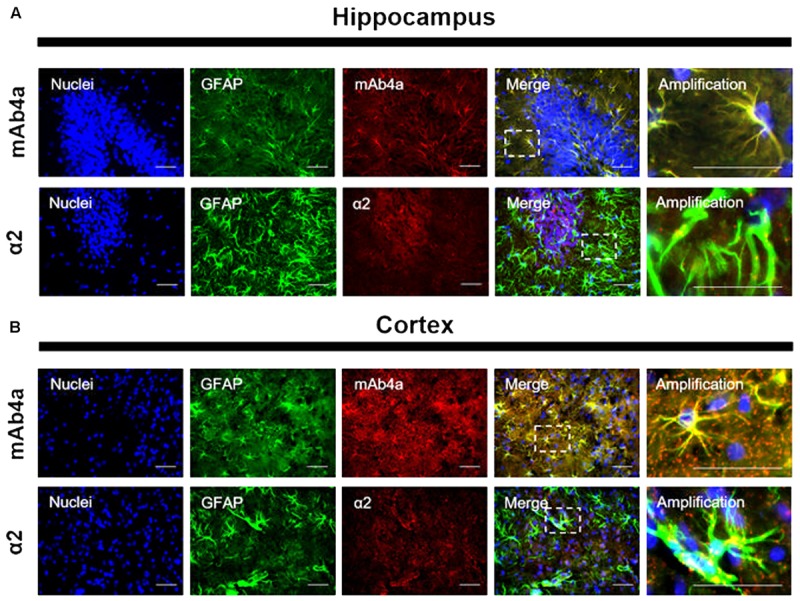
GlyR expression in rat brain astrocytes. Double detection of GFAP and mAb4a/α2 subunit in rat brain slices in **(A)** hippocampus and **(B)** cortex. Nuclei were stained with Hoechst, GFAP stained astrocytes are green and mAb4a/α2 immunoreactivity is red. Immunofluorescence images were acquired with a 40x objective in a Zeiss Axiovert 200. Dotted lines represent the amplified areas. Scale bars, 50 μm.

An immunohistochemistry assay in adult rat brain slices was performed to assess GlyR expression in rat brain astrocytes *in situ*. Slices (12 μm) were double labeled with an antibody against GFAP, to stain astrocytes, together with mAb4a or with the α2 subunit antibody. As shown in **Figure 5**, GlyR markers could be found in the cytoplasm and in the perinuclear space of GFAP-positive cells, in both hippocampus (**Figure 5A**) and cortex (**Figure 5B**). In both areas, mAb4a expression in GFAP-positive cells is higher than the expression of α2 subunit, indicating that GlyR is not a homomeric α2 receptor in mature astrocytes.

### GlyR Activation Inhibits ATP-Induced Calcium Transients in Astrocytes

**FIGURE 6:**
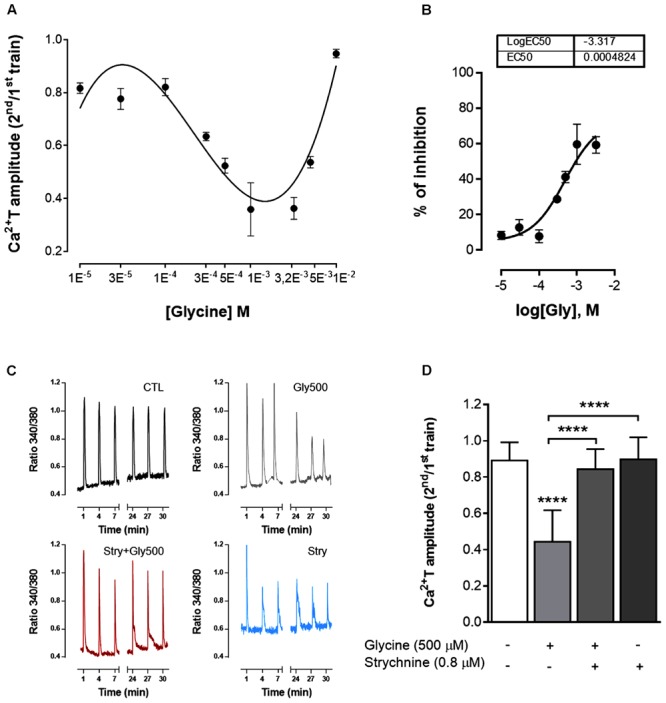
GlyR activation inhibits ATP-induced calcium transients in astrocytes. The biphasic response to increasing concentrations of glycine (abscissae) is shown in **(A)**, where the ordinates represent the averaged Ca^2+^T amplitude quantified as the ratio 2^nd^/1^st^ of the responses of ATP stimulation. In **(B)** is shown the log concentration-response curve for the first component of the glycine effect. Each point is mean ± SEM of the inhibitory effect of glycine obtained in *N* = 1–3 plates from independent cultures. **(C)** Representative plots of Ca^2+^T obtained in one experiment under control (CTL), glycine 500 μM (Gly500), strychnine 0.8 μM (Stry) and glycine + strychnine (Gly500 + Stry) conditions. **(D)** Summary plot of the amplitude of the responses to ATP (as ratio 2^nd^/1^st^), under the drug conditions indicated below each bar. All values are mean ± SEM, *n* = 33–42 responsive cells from 3 to 5 independent cultures. ^∗∗∗∗^*p* ≤ 0.0001, one-way ANOVA followed by Bonferroni’s Comparison Test. Statistical tests were performed in comparison with drug-free condition, except if otherwise indicated by the lines above the bars.

ATP (10 μM for 200 ms) caused a fast and transient cytosolic calcium rise (Ca^2+^T) in cultured astrocytes, as assessed by the calcium indicator, Fura 2 (**Figure 1**). To study the effect of drugs as modifiers of ATP-induced Ca^2+^T, two separated trains of ATP stimulation were performed, the first in the absence of drugs and used as an internal control, and the second in the presence of the test drugs. Drug effects were quantified as the ratio between the amplitudes of the ATP responses in the 2^nd^ over the 1^st^ train (2^nd^/1^st^, see Materials and Methods) and compared with the ratio obtained when both trains were delivered in drug-free conditions. In the experiments where glycine was present during the 2^nd^ train of stimulation, there was a clear decrease in the amplitude of Ca^2+^T in response to ATP, the 2^nd^/1^st^ ratio being lower than that obtained in drug free conditions (**Figure 6**). This inhibitory action of glycine was evident for concentrations higher than 100 μM, and reached a maximum around 3.2 mM of glycine (**Figures 6A,B**). At higher concentrations, the inhibitory effect of glycine was progressively lost (**Figure 6A**), suggestive of agonist-induced GlyR internalization. Non-linear regression analysis of the log concentration-response of the first component of the curve (**Figure 6B**), indicated an EC50 of 482 μM. Thus, all subsequent experiments carried out in this work were performed with 500 μM of glycine, a concentration also used by others to study the effect of GlyR activation in calcium signaling in oligodendrocyte progenitor cells (Belachew et al., 2000).

In the presence of 500 μM glycine (**Figures 6C,D**) the ratio 2^nd^/1^st^ (0.44 ± 0.030) was significantly lower (*p* < 0.0001, **Figure 6D**) than the one obtained in drug free conditions (0.89 ± 0.017). The effect of glycine was virtually abolished in the experiments where it was tested in the presence of the GlyR antagonist, strychnine 0.8 μM (**Figure 6D**). Strychnine 0.8 μM by itself did not affect the amplitude of Ca^2+^T (**Figure 6D**). These results indicate that glycine inhibits calcium signaling in astrocytes by activating GlyR.

### The Transducing System Operated by GlyR Is Similar to That Operated by Other Cl^-^ Permeant Receptors in Astrocytes

GlyR is a Cl^-^ channel and this ion was described as an intracellular messenger in astrocytes (Bekar and Walz, 1999).

The GABA_A_R is also a Cl^-^ channel, and is present in astrocytes (Bekar and Walz, 1999). Hypothesizing that Cl^-^ could be the mediator of the GlyR inhibitory effect upon the ATP-induced responses in astrocytes, we anticipated that GABA_A_R manipulation could yield a similar effect than GlyR activation. Thus, experiments were performed where GABA_A_R activity was manipulated, using a GABA_A_R agonist, muscimol, and a GABA_A_R antagonist, gabazine.

**FIGURE 7:**
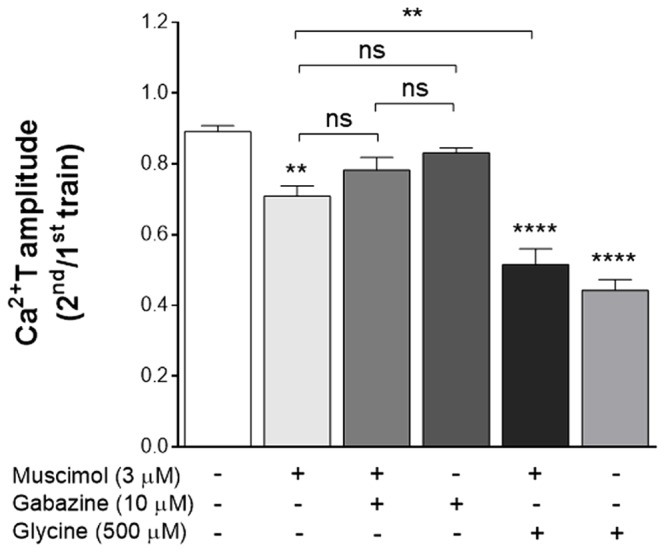
The inhibitory effect of glycine upon Ca^2+^T in astrocytes is mimicked by the GABA_A_R agonist muscimol. Data is represented as the averaged Ca^2+^T amplitude of the responses to ATP (as ratio 2^nd^/1^st^), under the drug conditions indicated below each bar. All values are mean ± SEM, *n* = 25–48 responsive cells from 3 to 5 independent cultures. ns, not significant, ^∗∗^*p* ≤ 0.01, ^∗∗∗∗^*p* ≤ 0.0001, one-way ANOVA followed by Bonferroni’s Comparison Test. Statistical tests were performed in comparison with drug-free condition, except if otherwise indicated by the lines above the bars. Representative curves for control, muscimol, muscimol + gabazine and gabazine alone are presented in **Supplementary Figure S1**.

When astrocytes were perfused with muscimol 3 μM, a concentration near three times its EC50 (Meyer et al., 2000), there was a significant decrease (**Figure 7**) in Ca^2+^T (ratio 2^nd^/1^st^: 0.71 ± 0.029, *p* < 0.01 vs drug free control). Representative tracings from these experiments are in **Supplementary Figure S1**. The effect of muscimol was lost in the presence of 10 μM gabazine (ratio 2^nd^/1^st^: 0.78 ± 0.036, *p* > 0.05 vs gabazine alone). Gabazine *per si* did not have any significant effect in Ca^2+^T (0.83 ± 0.015, *p* > 0.05, when compared to drug-free conditions). Remarkably, the effects of muscimol and glycine, added together, were not additive (**Figure 7**), suggesting a common transducing mechanism. Indeed, the effect of both drugs added together was lower (0.52 ± 0.044) than that of glycine alone (0.44 ± 0.030), indicative of a cross-inhibition between GlyR and GABA_A_R in astrocytes, as observed in neurons (Li et al., 2003).

Altogether these data suggest that Cl^-^ flux, through either GlyR or GABA_A_R channels, mediates an inhibitory effect upon ATP-induced Ca^2+^T in astrocytes.

### Glycine-Induced GlyR Translocation to the Plasma Membrane Is Required for Its Inhibitory Action upon Calcium Signaling

Based on our findings that in the absence of added glycine, GlyR were mostly confined to the cytosol (**Figures 4**, **5**) and on previous reports that GlyR traveling from the cytosol to the plasma membrane requires microtubule integrity via gephyrin binding (Hanus et al., 2004), we hypothesized that disruption of microtubule dynamics could affect the action of glycine, as well as GlyR location in astrocytes. To test this possibility we assessed the influence of nocodazole, an antimitotic agent that inhibits microtubule dynamics (Cotrina et al., 1998; Hanus et al., 2004), upon the action of glycine.

**FIGURE 8:**
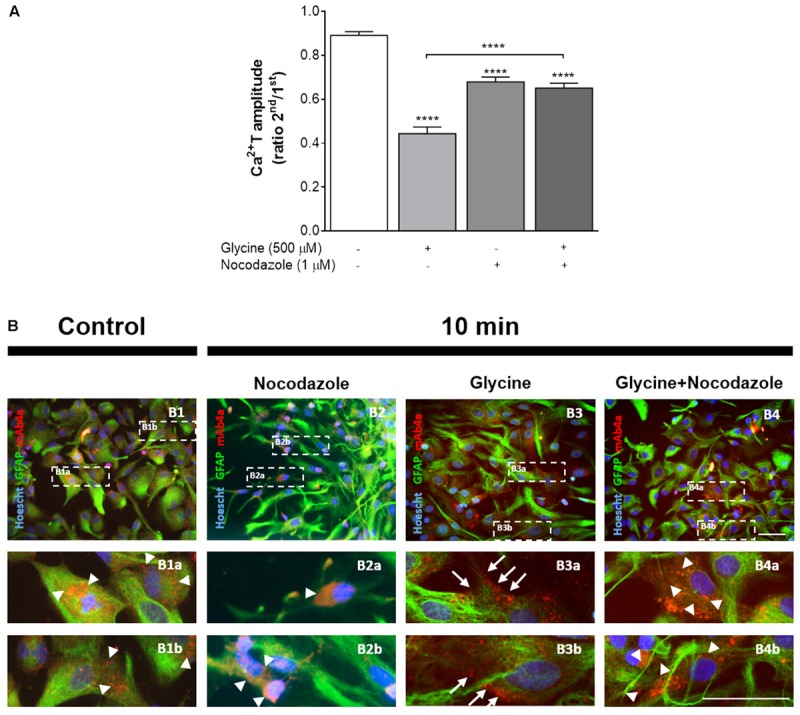
Impact of nocodazole, an inhibitor of microtubule dynamics, upon GlyR activation and localization. **(A)** Influence of nocodazole upon ATP-induced calcium transients and upon the effect of glycine. Data is represented as the averaged Ca^2+^T amplitude of the responses to ATP (as ratio 2^nd^/1^st^), under the drug conditions indicated below each bar. All values are mean ± SEM. *n* = 33–46 responsive cells from 3 to 5 independent cultures. ^∗∗∗∗^*p* ≤ 0.0001, one-way ANOVA followed by Bonferroni’s Comparison Test. Representative curves for Nocodazole and Nocodazole + Glycine can be found in **Supplementary Figure S2**. **(B)** Influence of nocodazole treatment in glycine (500 μM)-induced GlyR anchorage to the plasma membrane of astrocytes. Double detection of GlyR and GFAP in 14 DIV astrocytes, in drug-free conditions **(B1)**, and upon 10 min of incubation with nocodazole alone **(B2)**, glycine **(B3)** and glycine + nocodazole **(B4)**. Nuclei were stained with Hoechst, GFAP stained astrocytes are green and mAb4a immunoreactivity is red. Fluorescence images were acquired with a 40x objective in Zeiss Axiovert 200. Dotted lines indicate the amplified areas. Arrowheads indicate cytoplasmic GlyR, while arrows point to membrane bound GlyR. Scale bars, 50 μm.

Perfusion of astrocytes with nocodazole (1 μM), caused a significant reduction in Ca^2+^ transients amplitude (**Figure 8A**) when compared with the drug-free control (ratio 2^nd^/1^st^: 0.68 ± 0.022 vs 0.89 ± 0.017, *p* < 0.01), indicating that calcium signaling is sensitive to nocodazole treatment, as previously described (Cotrina et al., 1998). As also shown in **Figure 8A**, when glycine was tested in the presence of nocodazole it caused no further reduction in Ca^2+^T in relation to nocodazole alone (ratio 2^nd^/1^st^: 0.65 ± 0.022 vs 0.68 ± 0.022, *p* > 0.05). The ability of nocodazole to prevent the action of glycine cannot be solely attributed to the inhibitory action of nocodazole *per se* because glycine alone caused a greater inhibition of Ca^2+^T amplitude than nocodazole (**Figure 8A**). Representative curves of these experiments can be found in **Supplementary Figure S2**. Together, these data indicate that microtubule integrity is relevant for GlyR activation by glycine and consequent action upon calcium signaling in astrocytes.

We then hypothesized that the influence of nocodazole upon Ca^2+^T amplitude could result from impairment of GlyR membrane insertion. To address this possibility, the influence of nocodazole on GlyR localization within astrocytes was assessed by immunocytochemistry. Images show GlyR localization in a drug-free condition (**Figure 8B1**), and upon incubation for 10 min with nocodazole (**Figure 8B2**), glycine (500 μM) (**Figure 8B3**) and glycine together with nocodazole (**Figure 8B4**). It is clear that glycine induces GlyR recruitment to the cellular membrane of astrocytes (arrows in **Figures 8B3a,b**). In contrast, in the presence of nocodazole, glycine is no longer able to promote translocation of GlyR to the membrane, with GlyR being confined to the cytoplasm (arrowheads in **Figures 8B4a,b**), as observed in drug-free conditions (arrowheads in **Figures 8B1a,b**). Nocodazole *per se* does not affect astrocytic morphology nor GlyR expression within the cytoplasm (arrowheads in **Figures 8B2a,b**).

Overall, the results show that microtubule-dependent glycine-mediated GlyR clustering at the plasma membrane is a crucial step for GlyR inhibitory action upon calcium signaling in astrocytes.

## Discussion

**FIGURE 9:**
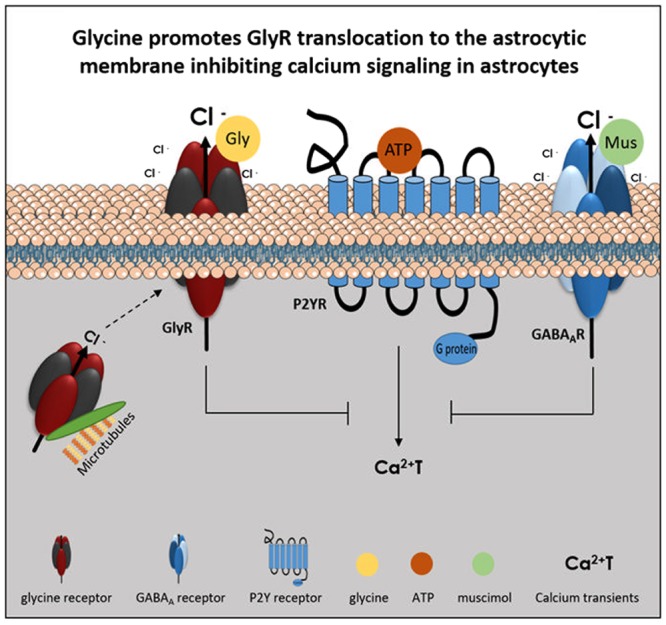
Model of GlyR activation effect upon ATP-induced calcium transients in astrocytes. ATP caused an increase in cytosolic calcium (Ca^2+^T) by P2Y receptor (P2YR) stimulation. Glycine receptor (GlyR) activation, by glycine (Gly), causes Cl^-^ efflux and leads to an inhibition of ATP-induced calcium transients. The effect is mimicked by activation of another Cl^-^-permeant receptor as the muscimol (Mus)-sensitive GABA_A_ receptor (GABA_A_R). GlyR anchorage at the plasma membrane, through a microtubule dependent transport, is necessary for GlyR activation by glycine and its effect upon astrocytic calcium signaling.

The main findings of the present work are the expression of GlyR in brain astrocytes and the inhibitory effect of GlyR activation upon astrocytic Ca^2+^T. We show that GlyR, predominantly subunits α2 and β, are expressed in brain astrocytes within the cytoplasm and astrocytic processes. Our data demonstrate, for the first-time, that GlyR activation by glycine leads to a decrease in Ca^2+^T amplitude in astrocytes. This inhibitory effect is mediated by Cl^-^ ions and requires GlyR anchorage at the plasma membrane, through a microtubule-dependent transport (**Figure 9**).

GlyR expression in astrocytes was previously demonstrated in rat spinal cord slices (Pastor et al., 1995; Kirchhoff et al., 1996). The results herein reported provide evidence that GlyR is expressed in brain astrocytes and indicate that GlyR is composed of GlyR α2 and β subunits. Interestingly, our results differ from those in spinal cord glial cells where only evidence of α1 and β subunits expression was found (Kirchhoff et al., 1996). Furthermore, we show that mature astrocytes express GlyR in the cytoplasm and in the perinuclear space, which is in accordance with studies performed in neurons (Hanus et al., 2004).

Exciting findings have been made regarding astrocytes during the past 15 years, which have led many researchers to redefine how the brain works. Astrocytes are now widely regarded as cells that propagate Ca^2+^ over long distances in response to stimulation, and release gliotransmitters in a Ca^2+^-dependent manner. However, how this Ca^2+^ signaling is negatively modulated is still poorly understood. In this work, calcium imaging assays were performed to assess the role of GlyR, an inhibitory receptor in mature neurons, upon Ca^2+^ signaling in astrocytes. The perfusion of cultured astrocytes with glycine 500 μM decreased ATP-induced Ca^2+^T, being the effect reversed by the presence of the GlyR antagonist strychnine. At the concentration used, 0.8 μM, strychnine is highly selective for GlyR, indicating that the observed effect was due to GlyR activation (Chattipakorn and McMahon, 2003; Li et al., 2003). Glycine application in neurons and oligodendrocytes precursors showed a similar inhibitory effect and was also associated with GlyR activation (Belachew et al., 2000; Bowery and Smart, 2006; Xu and Gong, 2010).

Chloride is the most abundant biological anion and was previously described as an intracellular messenger in astrocytes (Bekar and Walz, 1999, 2002; Walz, 2002). Its concentration was reduced in the cytoplasm of astrocytes after GABA_A_R activation (Bekar and Walz, 2002) and in microglia this ion can control internal store operated calcium entrance (McLarnon et al., 2000). The study of Cl^-^ Intracellular Ion Channels (CLICs) developed over the past two decades, but only recently such channels were described and analyzed in astrocytes and much is still unknown (Walz, 2002; Kimelberg et al., 2006). Several roles for these channels were proposed, including acidification of intracellular compartments and release of calcium from endoplasmic and sarcoplasmic reticuli, among others (Edwards and Kahl, 2010). As GlyR activation, we herein show that GABA_A_R activation, by muscimol (3 μM), decreases Ca^2+^T, which together with the knowledge that both receptors in neurons are permeant to Cl^-^ suggests a similar transducing pathway involving Cl^-^ movement across the plasma membrane. In astrocytes, due to the direction of the electrochemical gradient, activation of GABA_A_R has been shown to induce Cl^-^ efflux (Bekar and Walz, 2002). It is thus likely that GlyR activation in astrocytes also promotes Cl^-^ efflux, thus decreasing the intracellular concentration of Cl^-^, and modulating the activity of a CLIC known to regulate cellular Ca^2+^ homeostasis (Pollock et al., 1998). However, if a CLIC is involved the exact mechanism by which it operates is still unknown.

As discussed above GABA_A_R activation has an inhibitory effect in Ca^2+^T, although less pronounced than GlyR activation. When the two agonists (glycine 500 μM + muscimol 3 μM) were perfused together their effects were not cumulative. The same findings have been previously reported in neurons, which show a state-dependent cross-inhibition between these two receptors. In these studies, GlyR activation negatively modulates GABA_A_R, resulting in a depressed GABA-mediated response. The opposite result has also been shown, with a GlyR-mediated depressed response under GABA_A_R activation (Li and Xu, 2002; Li et al., 2003). In this state-dependent cross-inhibition, GlyR activation triggers intracellular phosphorylation pathways that lead to phosphatase 2B activation and alterations in GABA_A_R conformation. Subsequently, GABA_A_R conformational change can lead to exposure of receptor phosphorylation sites, where phosphatase 2B can act. In turn, GlyR inhibition by GABA_A_R activation is suggested to occur by coupling between the two receptors (Li et al., 2003). Indeed, GlyR-GABA_A_R complexes have already been demonstrated in the brain (Li and Xu, 2002). Considering our results, it would be interesting to evaluate if the same mechanisms are present in astrocytes.

GlyR and GABA_A_R require gephyrin for a proper anchoring at the synaptic membrane, and receptor recruitment to the plasma membrane is microtubule dependent, and thus sensible to nocodazole treatment (Hanus et al., 2004). Nocodazole itself caused a consistent decrease of the Ca^2+^T elicited by ATP. However, we observed that the joint perfusion of glycine and nocodazole did not further decrease Ca^2+^T in relation to nocodazole perfusion alone, which pointed to a direct effect of nocodazole over GlyR recruitment to the membrane. To further prove this hypothesis, we evaluated GlyR cellular localization in the presence of glycine alone and with nocodazole, and have found that, in the presence of glycine, GlyR was recruited to the plasma membrane, whereas in the simultaneous presence of glycine and nocodazole GlyR was confined to the cytoplasm. These results pointed to the need of intact microtubules for GlyR recruitment to the plasma membrane of astrocytes, which corroborates with the reported in neurons. Indeed, in neurons, nocodazole treatment decreases the rate of GlyR accumulation at the cellular membrane and reduces the GlyR-gephyrin small aggregates along the cytoplasm, indicating that the stabilization of the receptor in the membrane was gephyrin dependent and required microtubules (Hanus et al., 2004; Avila et al., 2013).

## Conclusion

We herein demonstrate that GlyR is expressed in brain astrocytes and that its activation negatively modulates calcium signaling in these cells. These data thus highlight a novel role for glycine in the nervous system controlling astrocytic signaling and being part of a mechanism to modulate astrocyte-to-neuron communication.

## Author Contributions

CV and AS conceived the project. All authors designed the experiments and revised it. TM performed the molecular-based techniques. TM and DC carried out the calcium imaging assays. SV provided supervision in the calcium imaging assays. CV and AS provided substantial contributions to the interpretation of data. TM wrote the manuscript. All authors read and approved the final version.

## Conflict of Interest Statement

The authors declare that the research was conducted in the absence of any commercial or financial relationships that could be construed as a potential conflict of interest.

## Supplementary Material

**FIGURE S1 |** Representative responses to ATP in an experiment under control (CTL) conditions, and in the presence of muscimol, muscimol + gabazine and gabazine alone during the 2^nd^ train of stimulation.

**FIGURE S2 |** Representative responses to ATP in an experiment where nocodazole (Noc) or nocodazole + glycine (Noc + Gly500) were present during the 2^nd^ train of stimulation.

## References

[B1] Allaman I. Bélanger M. Magistretti P. J. (2011). Astrocyte–neuron metabolic relationships: for better and for worse. *Trends Neurosci.* 34 76–87. 10.1016/j.tins.2010.12.001 21236501

[B2] Allen N. J. Barres B. A. (2009). Neuroscience: Glia - more than just brain glue. *Nature* 457 675–677. 10.1038/457675a 19194443

[B3] Araque A. Parpura V. Sanzgiri R. P. Haydon P. G. (1999). Tripartite synapses: glia, the unacknowledged partner. *Trends Neurosci.* 22 208–215. 10.1016/S0166-2236(98)01349-6 10322493

[B4] Aroeira R. I. Ribeiro J. A. Sebastião A. M. Valente C. A. (2011). Age-related changes of glycine receptor at the rat hippocampus: from the embryo to the adult. *J. Neurochem.* 118 339–353. 10.1111/j.1471-4159.2011.07197.x 21272003

[B5] Aroeira R. I. Sebastião A. M. Valente C. A. (2014). GlyT1 and GlyT2 in brain astrocytes: expression, distribution and function. *Brain Struct. Funct.* 219 817–830. 10.1007/s00429-013-0537-3 23529192

[B6] Aroeira R. I. Sebastião A. M. Valente C. A. (2015). BDNF, via truncated TrkB receptor, modulates GlyT1 and GlyT2 in astrocytes. *Glia* 63 2181–2197. 10.1002/glia.22884 26200505

[B7] Avila A. Nguyen L. Rigo J.-M. (2013). Glycine receptors and brain development. *Front. Cell. Neurosci.* 7:184 10.3389/fncel.2013.00184 PMC3800850 24155690

[B8] Barres B. A. (2008). The mystery and magic of glia: a perspective on their roles in health and disease. *Neuron* 60 430–440. 10.1016/j.neuron.2008.10.013 18995817

[B9] Bekar L. K. Walz W. (1999). Evidence for chloride ions as intracellular messenger substances in astrocytes. *J. Neurophysiol.* 82 248–254. 10400953 10.1152/jn.1999.82.1.248

[B10] Bekar L. K. Walz W. (2002). Intracellular chloride modulates a-type potassium currents in astrocytes. *Glia* 39 207–216. 10.1002/glia.10096 12203387

[B11] Belachew S. Malgrange B. Rigo J. M. Rogister B. Leprince P. Hans G. (2000). Glycine triggers an intracellular calcium influx in oligodendrocyte progenitor cells which is mediated by the activation of both the ionotropic glycine receptor and Na^+^ - dependent transporters. *Eur. J. Neurosci.* 12 1924–1930. 10886333 10.1046/j.1460-9568.2000.00085.x

[B12] Bowery N. G. Smart T. G. (2006). GABA and glycine as neurotransmitters: a brief history. *Br. J. Pharmacol.* 147(Suppl.), S109–S119. 10.1038/sj.bjp.0706443 16402094 PMC1760744

[B13] Burnstock G. Krügel U. Abbracchio M. P. Illes P. (2011). Purinergic signalling: from normal behaviour to pathological brain function. *Prog. Neurobiol.* 95 229–274. 10.1016/j.pneurobio.2011.08.006 21907261

[B14] Chattipakorn S. C. McMahon L. L. (2003). Strychnine-sensitive glycine receptors depress hyperexcitability in rat dentate gyrus. *J. Neurophysiol.* 89 1339–1342. 10.1152/jn.00908.2002 12612034

[B15] Cotrina M. L. Lin J. H. Nedergaard M. (1998). Cytoskeletal assembly and ATP release regulate astrocytic calcium signaling. *J. Neurosci.* 18 8794–8804. 9786986 10.1523/JNEUROSCI.18-21-08794.1998 PMC6793564

[B16] Danglot L. Rostaing P. Triller A. Bessis A. (2004). Morphologically identified glycinergic synapses in the hippocampus. *Mol. Cell. Neurosci.* 27 394–403. 10.1016/j.mcn.2004.05.007 15555918

[B17] Dutertre S. Becker C.-M. Betz H. (2012). Inhibitory glycine receptors: an update. *J. Biol. Chem.* 287 40216–40223. 10.1074/jbc.R112.408229 23038260 PMC3504737

[B18] Edwards J. C. Kahl C. R. (2010). Chloride channels of intracellular membranes. *FEBS Lett.* 584 2102–2111. 10.1016/j.febslet.2010.01.037 20100480 PMC2929963

[B19] Eulenburg V. (2011). S.28.01 Glycine transporters: essential regulators of synaptic transmission. *Eur. Neuropsychopharmacol.* 21:S230 10.1016/S0924-977X(11)70352-2 16417482

[B20] Eulenburg V. Armsen W. Betz H. Gomeza J. (2005). Glycine transporters: essential regulators of neurotransmission. *Trends Biochem. Sci.* 30 325–333. 10.1016/j.tibs.2005.04.004 15950877

[B21] Fellin T. Carmignoto G. (2004). Neurone-to-astrocyte signalling in the brain represents a distinct multifunctional unit. *J. Physiol.* 559 3–15. 10.1113/jphysiol.2004.063214 15218071 PMC1665073

[B22] Gage G. J. Kipke D. R. Shain W. (2012). Whole animal perfusion fixation for rodents. *J. Vis. Exp.* 65:3564. 10.3791/3564 22871843 PMC3476408

[B23] García-Alcocer G. Mejía C. Berumen L. C. Miledi R. Martínez-Torres A. (2008). Developmental expression of glycine receptor subunits in rat cerebellum. *Int. J. Dev. Neurosci.* 26 319–322. 10.1016/j.ijdevneu.2008.01.005 18339511

[B24] Hanus C. Vannier C. Triller A. (2004). Intracellular association of glycine receptor with gephyrin increases its plasma membrane accumulation rate. *J. Neurosci.* 24 1119–1128. 10.1523/JNEUROSCI.4380-03.2004 14762130 PMC6793588

[B25] Jacob P. F. Vaz S. H. Ribeiro J. A. Sebastião A. M. (2014). P2Y_1_ receptor inhibits GABA transport through a calcium signalling-dependent mechanism in rat cortical astrocytes. *Glia* 62 1211–1226. 10.1002/glia.22673 24733747

[B26] Johnson J. W. Ascher P. (1987). Glycine potentiates the NMDA response in cultured mouse brain neurons. *Nature* 325 529–531. 10.1038/325529a0 2433595

[B27] Kimelberg H. K. MacVicar B. A. Sontheimer H. (2006). Anion channels in astrocytes: biophysics, pharmacology, and function. *Glia* 54 747–757. 10.1002/glia.20423 17006903 PMC2556042

[B28] Kirchhoff F. Mülhardt C. Pastor A. Becker C.-M. Kettenmann H. (1996). Expression of glycine receptor subunits in glial cells of the rat spinal cord. *J. Neurochem.* 66 1383–1390. 10.1046/j.1471-4159.1996.66041383.x 8627289

[B29] Langosch D. Thomas L. Betz H. (1988). Conserved quaternary structure of ligand-gated ion channels: the postsynaptic glycine receptor is a pentamer. *Proc. Natl. Acad. Sci. U.S.A.* 85 7394–7398. 10.1073/pnas.85.19.7394 2459705 PMC282193

[B30] Li Y. Wu L.-J. Legendre P. Xu T.-L. (2003). Asymmetric cross-inhibition between GABAA and glycine receptors in rat spinal dorsal horn neurons. *J. Biol. Chem.* 278 38637–38645. 10.1074/jbc.M303735200 12885784

[B31] Li Y. Xu T. L. (2002). State-dependent cross-inhibition between anionic GABAA and glycine ionotropic receptors in rat hippocampal CA1 neurons. *Neuroreport* 13 223–226. 11893914 10.1097/00001756-200202110-00010

[B32] McLarnon J. G. Helm J. Goghari V. Franciosi S. Choi H. B. Nagai A. (2000). Anion channels modulate store-operated calcium influx in human microglia. *Cell Calcium* 28 261–268. 10.1054/ceca.2000.0150 11032781

[B33] Meyer D. K. Olenik C. Hofmann F. Barth H. Leemhuis J. Brünig I. (2000). Regulation of somatodendritic GABA_A_ receptor channels in rat hippocampal neurons: evidence for a role of the small GTPase Rac1. *J. Neurosci.* 20 6743–6751. 10995817 10.1523/JNEUROSCI.20-18-06743.2000 PMC6772837

[B34] Nakahata Y. Eto K. Murakoshi H. Watanabe M. Kuriu T. Hirata H. (2017). Activation-dependent rapid postsynaptic clustering of glycine receptors in mature spinal cord neurons. *eNeuro* 4:ENEURO.0194-16.2017. 10.1523/ENEURO.0194-16 28197549 PMC5292596

[B35] Oberheim N. A. Goldman S. A. Nedergaard M. (2012). Heterogeneity of astrocytic form and function. *Methods Mol. Biol.* 814 23–45. 10.1007/978-1-61779-452-0_3 22144298 PMC3506190

[B36] Orellana J. A. Stehberg J. (2014). Hemichannels: new roles in astroglial function. *Front. Physiol.* 5:193 10.3389/fphys.2014.00193 PMC4060415 24987373

[B37] Pastor A. Chvatal A. Sykova E. Kettenmann H. (1995). Glycine- and GABA-activated currents in identified glial cells of the developing rat spinal cord slice. *Eur. J. Neurosci.* 7 1188–1198. 10.1111/j.1460-9568.1995.tb01109.x 7582092

[B38] Perea G. Araque A. (2005). Glial calcium signaling and neuron–glia communication. *Cell Calcium* 38 375–382. 10.1016/j.ceca.2005.06.015 16105683

[B39] Perea G. Araque A. (2010). GLIA modulates synaptic transmission. *Brain Res. Rev.* 63 93–102. 10.1016/j.brainresrev.2009.10.005 19896978

[B40] Perea G. Navarrete M. Araque A. (2009). Tripartite synapses: astrocytes process and control synaptic information. *Trends Neurosci.* 32 421–431. 10.1016/j.tins.2009.05.001 19615761

[B41] Pfaffl M. (2001). A new mathematical model for relative quantification in real-time RT-PCR. *Nucleic Acids Res.* 29:e45. 10.1093/nar/29.9.e45 PMC55695 11328886

[B42] Pollock N. S. Kargacin M. E. Kargacin G. J. (1998). Chloride channel blockers inhibit Ca2+ uptake by the smooth muscle sarcoplasmic reticulum. *Biophys. J.* 75 1759–1766. 10.1016/S0006-3495(98)77617-9 9746517 PMC1299847

[B43] Sofroniew M. V. (2010). Molecular dissection of reactive astrogliosis and glial scar formation. *Trends Neurosci.* 32 638–647. 10.1016/j.tins.2009.08.002.Molecular PMC2787735 19782411

[B44] Tyagarajan S. K. Fritschy J.-M. (2014). Gephyrin: a master regulator of neuronal function? *Nat. Rev. Neurosci.* 15 141–156. 10.1038/nrn3670 24552784

[B45] Volterra A. Liaudet N. Savtchouk I. (2014). Astrocyte Ca(2+) signalling: an unexpected complexity. *Nat. Rev. Neurosci.* 15 327–335. 10.1038/nrn3725 24739787

[B46] Volterra A. Steinhäuser C. (2004). Glial modulation of synaptic transmission in the hippocampus. *Glia* 15 249–257. 10.1002/glia.20080 15252814

[B47] Walz W. (2002). Chloride/anion channels in glial cell membranes. *Glia* 40 1–10. 10.1002/glia.10125 12237839

[B48] Wang D. D. Bordey A. (2008). The astrocyte odyssey. *Prog. Neurobiol.* 86 342–367. 10.1016/j.pneurobio.2008.09.015 18948166 PMC2613184

[B49] Xu T.-L. Gong N. (2010). Glycine and glycine receptor signaling in hippocampal neurons: diversity, function and regulation. *Prog. Neurobiol.* 91 349–361. 10.1016/j.pneurobio.2010.04.008 20438799

