# Supplementary figures and images for: Glycine Receptor Activation Impairs ATP-Induced Calcium Transients in Cultured Cortical Astrocytes
Source: Front Mol Neurosci. 2018 Jan 17;10:444. doi: 10.3389/fnmol.2017.00444 (PMC5776331; doi:10.3389/fnmol.2017.00444)

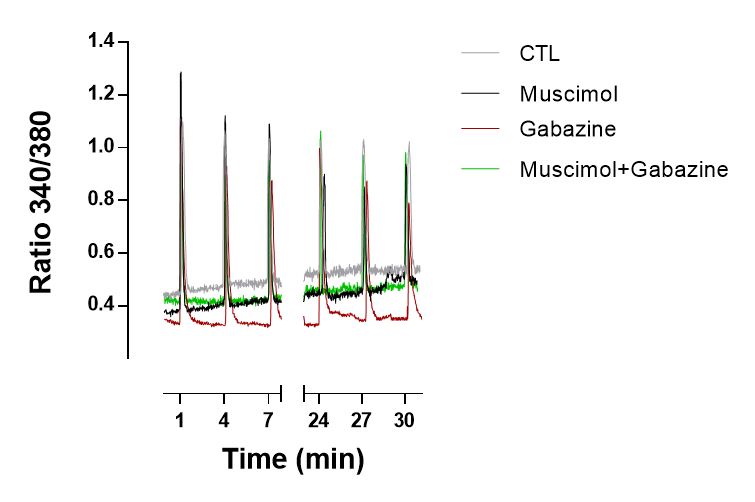

Supplement: Supplementary file 1 [file Image_1.TIF]

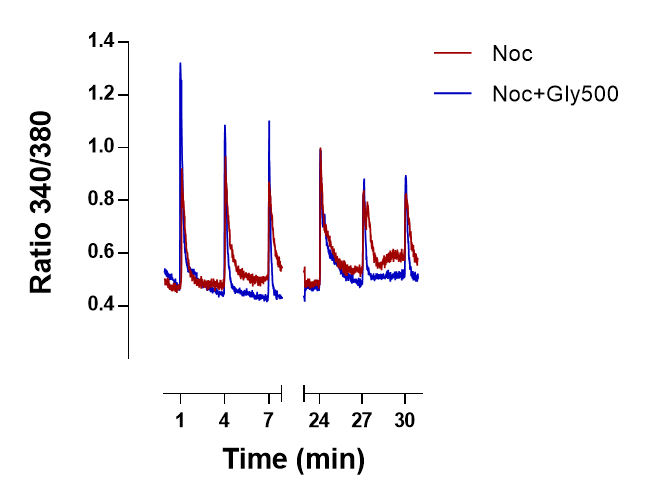

Supplement: Supplementary file 2 [file Image_2.TIF]
